# Hypoxia-induced RBBP7 promotes esophagus cancer progression by inducing CDK4 expression: RBBP7 promotes esophagus cancer progression

**DOI:** 10.3724/abbs.2021027

**Published:** 2022-01-19

**Authors:** Renfeng Wang, Zhiliang Huang, Zhenyang Lin, Borong Chen, Xuguang Pang, Chaoxiang Du, Hong Fan

**Affiliations:** Department of Thoracic Surgery Zhongshan Hospital Fudan University (Xiamen Branch) Xiamen 361015 China

**Keywords:** esophagus cancer, RBBP7, hypoxia, HIF1α, CDK4, tumor stemness

## Abstract

Hypoxia-induced epigenetic regulation calls for more effective therapeutic targets for esophageal cancer. We used GEPIA and UALCAN databases to screen survival-related and cancer stage-associated genes. Eca109 and KYSE450 esophageal cancer cell lines were cultured under normoxia, hypoxia, or CoCl _2_-induced hypoxia conditions, which were further transfected with plasmids expressing RB binding protein 7 (RBBP7), hypoxia-inducible factor 1 (HIF1)-α, or RBBP7 shRNA. Colony formation and MTT assays were used to detect cell proliferation. Tumor sphere formation and stemness marker detection were applied to assess cell stemness. RT-PCR and western blot analysis were used to detect the relative mRNA level and protein expression, respectively. Luciferase assay was utilized to detect the direct interaction between HIF1α and RBBP7. Up-regulated RBBP7 was identified as one of the most prominent survival-related genes, which is negatively correlated with the overall survival (OS), disease recurrence-free survival (DFS), and tumor stages. Hypoxia-induced HIF1α up-regulates RBBP7 expression, which promotes esophagus cancer cell viability, proliferation, and stemness with increased cyclin-dependent kinase 4 (CDK4) expression. Luciferase reporter assay verified that HIF1α transcriptionally regulates the expression of RBBP7. We conclude that hypoxia induces high expression of RBBP7 which is at least partially mediated by HIF1α, up-regulates the expression of downstream CDK4, and thereby promotes tumor progression in esophageal cancer cells.

## Introduction

Esophageal cancer, one of the most common cancers worldwide, is still one of the most clinically challenging malignancies [1]. Although chemotherapy, radiation therapy, surgery, and a combination of the above methods are available for cancer treatment [2], the five-year survival rate for esophageal cancer is only 20%–30% [ 3, 4]. Hypoxia, a common feature of advanced esophageal cancer, is involved in chemoresistance and radiotherapy resistance response, which may utilize mechanisms including epigenetic alteration and chromatin remodeling, such as DNA methylation and histone modifications, to promote malignancy transformation [ 5, 6]. A profound understanding of hypoxia-induced epigenetic alterations will broaden the understanding of more effective therapeutic targets for esophageal cancer.

Retinoblastoma-binding protein 7 (RBBP7) is a core component of many complexes for chromatin remodeling and histone modification [7], which is overexpressed in many kinds of cancers and exerts conflicting roles in tumor progression. RBBP7 can inhibit transcriptional trans-activation mediated by breast cancer type 1 (BRCA1) to affect cell proliferation and differentiation [8]. In neoplastigenic breast epithelial cells, stress-induced apoptosis is promoted by overexpression of RBBP7, and tumorigenicity is suppressed [9]. In mammary epithelial cells, the epithelial-mesenchymal transition is induced by the constitutive expression of RBBP7 [10]. In contrast, little research has been performed to decipher the role of RBBP7 in esophageal cancer.

Uncontrolled cell growth and proliferation are the hallmarks of cancer [ 11, 12]. Cyclin-dependent kinases (CDKs), which belong to a family of serine/threonine (Ser/Thr) protein kinases, play a key role in regulating the cell cycle [ 13, 14]. Many epigenetic and genetic events affect CDK, result in the loss of checkpoint integrity, and promote cell proliferation and malignant transformation. Some studies have verified that CDK4 is usually overexpressed and/or over-actived in human esophageal cancer [ 15, 16], which can regulate the G1-S phase of the cell cycle by inactivating the tumor-suppressive retinoblastoma protein (Rb) in cancer cells and dividing cells [17]. Therefore deciphering the association between CDK4 and RBBP7 may provide a promising method for cancer treatment.

Currently, there are relatively few reports on RBBP7 in esophageal cancer. We hereby found that RBBP7 could promote the proliferation of esophageal cancer cells and increase stemness. Mechanistic analysis revealed that hypoxia-induced HIF1α could up-regulate RBBP7 to promote the expression of downstream CDK4, thereby enabling the tumor progression of esophageal cancer cells.

## Materials and Methods

### Cell culture, transfection and cell viability assays

Eca109 and KYSE450 esophageal cancer cell lines (ATCC, Manassas, USA) were cultured in Dulbecco’s modified Eagle’s medium (DMEM; Gibco, Grand Island, USA) with 10% fetal bovine serum (FBS; Gibco), 10,000 U/mL penicillin, and 10 μg/mL streptomycin. The cells were cultured at 37°C in humidified atmosphere containing 5% CO _2_. The Eca109 and KYSE450 cell lines were authenticated by STR genotyping ( Supplementary Figure S1). Cell viability was assessed by 3-(4,5-dimethylthiazol-2-yl)-2,5-diphenyltetrazolium bromide (MTT) assay.

Briefly, the cells were seeded onto 96-well plates at 2×10 ^3^ cells/well and cultured for 24 h. Then the plasmids of RBBP7 (#176411; Addgene, Watertown, USA) and HIF1α (#163365; Addgene) were transfected using Lipofectamine™ 3000 (Thermo Fisher Scientific, Waltham, USA). The cells were treated with 10 μL MTT solution (5 mg/mL; Sigma-Aldrich, St Louis, USA) for 4 h at different time points. After removal of the supernatant, 100 μL of DMSO was added to dissolve the blue formazan crystals. Plates were scanned at 595 nm and 650 nm using a Multiskan Spectrum spectrophotometer (Thermo Fisher Scientific). Data were presented as the percentage of the control (% control).

### Cell colony formation assay

The cells were seeded at 200 cells/well in a six-well plate and transfected with RBBP7 plasmids (#176411; Addgene) or control vectors. After 2 weeks of culture, the cell colonies emerged. Phosphate-buffered saline (PBS) was used to gently wash the plate for three times. Then, methanol was applied to fix the cell colonies, and finally the colonies were stained with 0.5% Giemsa (Beyotime, Shanghai, China). The number of colonies from each well was counted under an inverted microscope (TS100; Nicon, Tokyo, Japan) and authenticated by their karyotypes and morphologies.

### RNA extraction and quantitative RT-PCR

Total RNA was extracted from Eca109 and KYSE450 esophageal cancer cells using Trizol reagent (Thermo Fisher Scientific), reverse-transcribed into complementary DNA (cDNA) using PrimeScript First Strand cDNA synthesis kit (Takara, Shiga, Japan) according to the manufacturer’s instructions. Quantitative PCR was performed (95°C for 30 s; 40 cycles at 95°C for 10 s; 52°C for 10 s; and 72°C for 10 s) using SYBR Green Premix Ex Taq (Takara) to determine the mRNA expression of target genes. The relative expression was normalized to 18S RNA. The primers used were as follows: *RBBP7* forward 5′-ATGGCGAGTAAAGAGATGTT-3′, reverse 5′-TTAAGATCCTTGTCCCTCCA-3′; *HIF1α* forward 5′-ACCTTCATCGGAAACTCCAAAG-3′, reverse 5′-CTGTTAGGCTGGGAAAAGTTAGG-3′; 18S forward 5′-GTAACCCGTTGAACCCCATT-3′, reverse 5′-CCATCCAATCGGTAGTAGCG-3′. The relative expression of each gene was calculated with the 2 ^−ΔΔCt^ method.

### Tumor sphere formation assay

The Eca109 and KYSE450 cells were harvested and re-suspended as single cells in serum-free DMEM/F12 medium (Invitrogen, Waltham, USA) supplemented with B27 (1:50; Gibco), 100 U/mL penicillin, 100 μg/mL streptomycin, 4 μg/mL heparin (Sigma-Aldrich), 20 ng/mL EGF (Pepro Tech, Bedford, USA), and 20 ng/mL bFGF (Pepro Tech). After accurate cell counting, 200 cells in 200 μL of serum-free DMEM/F12 medium were added into each well of a 96-well plate, with 10 wells in each group. The medium was changed every two days. After seven days, cell morphology was examined under a light microscopy (LV100N; Nicon). Images of five randomly selected regions of each group were taken with a phase-contrast microscope (DMi1; Leica, Wetzlar, Germany). The sphere formation was calculated as the number of spheres generated from the number of cells seeded (200 cells).

### Cell treatmet

Eca109 and KYSE450 esophageal cancer cell lines were cultured at 2×10 ^6^ cells/mL in six-well plates in DMEM with 10% FBS and antibiotics (10,000 U/mL penicillin and 10 μg/mL streptomycin) at 37°C in a humidified atmosphere containing 5% CO _2_ (normoxia) or in a hypoxial tank containing 94% N _2_, 5% CO _2_ and 1% O _2_ (hypoxia) for 4 h. Then cells were harvested for RT-PCR and western blot analysis. For CoCl _2_ treatment, Eca109 and KYSE450 cells were cultured in DMEM with or without 200 μM CoCl _2_ (Sigma-Aldrich) for 48 h prior to RT-PCR and western blot analysis.

### Luciferase assay

The 3′UTR of HIF1α (GenScript, Nanjing, China) containing RBBP7 binding site was cloned into the downstream of the luciferase coding region in the pmirGLO vector (GenScript). The sequence of HIF1α 3′UTR containing the binding sites of RBBP7 was mutated. The cells were co-transfected with wild-type or mutant luciferase reporter constructs. After 48 h of transfection, the activity of luciferase constructs was measured using a DualGlo Luciferase Assay System (Promega) according to the manufacturer’s instructions. The Renilla luciferase activity was used for normalization.

### Western blot analysis

Eca109 and KYSE450 cells were collected, and the total protein was extracted using RIPA Lysis Buffer (Thermo Fisher Scientific) and separated on 10% SDS-PAGE gels. After being transferred to PVDF membranes, 5% non-fat milk was used to block the membranes at room temperature for 1 h. Then, membranes were incubated with primary antibodies (RBBP7, CDK4; 1:1000; Millipore, Billerica, USA) at 4°C overnight, followed by incubation with the corresponding HRP-conjugated secondary antibody (Invitrogen) at room temperature for 1 h. The protein bands were detected using an enhanced chemiluminescence detection systems (Bio-Rad, Hercules, USA), and the gray scale of the bands was analyzed by ImageJ software.

### Statistics analysis

All data were expressed as the mean ± SD. SPSS software was used for statistical analysis. One-way analysis of variance (ANOVA) followed by a post hoc test or Student’s *t*-test was applied for comparisons between groups. *P* value of less than 0.05 was considered statistically significant.

## Results

### RBBP7 correlates with poor survival outcomes in esophagus cancer

**Figure 1:**
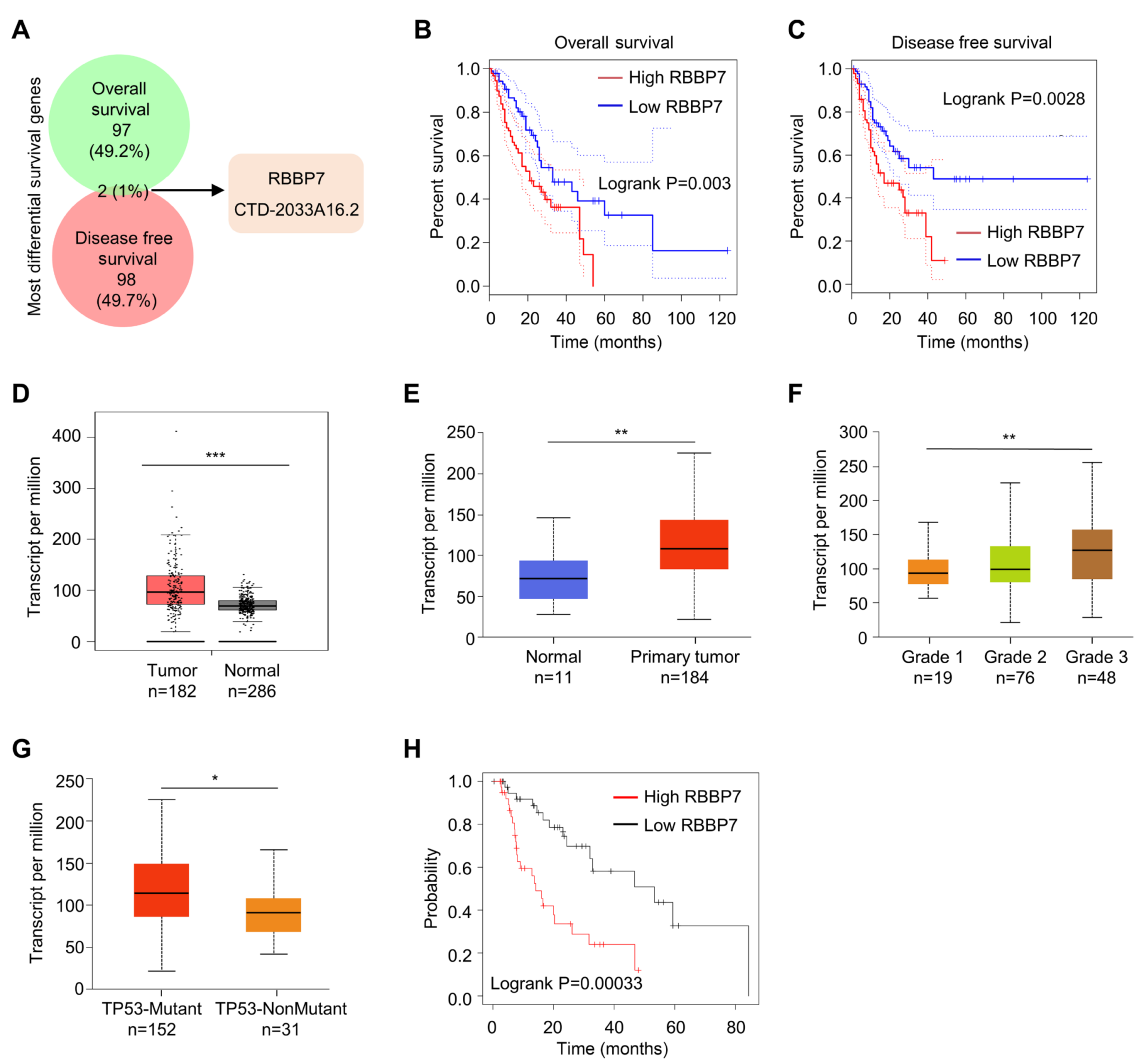
Figure1 **RBBP7 correlates with poor survival outcomes in esophagus cancer**(A) Venn diagram shows the intersection set of Most Differential Survival Genes in esophagus cancer patients in GEPIA datasets. (B) Kaplan-Meier analysis of overall survival in esophagus cancer patients according to different RBBP7 levels in GEPIA datasets. (C) Kaplan-Meier analysis of disease recurrence-free survival in esophagus cancer patients according to different RBBP7 levels in GEPIA datasets. (D) Tumor tissues (182 from TCGA) and 286 normal tissues (from GTEx) were analyzed to compare the expression level of RBBP7 mRNA. (E) Expression of RBBP7 in normal and esophagus cancer tissues in TCGA datasets. (F) Expression of RBBP7 in esophagus cancer patients with different tumor stages in TCGA datasets. (G) Expression of RBBP7 in esophagus cancer patients with different TP53 status in TCGA datasets. (H) Kaplan–Meier plots of overall survival in esophagus cancer patients stratified according to their RBBP7 levels in TCGA datasets. Data are shown as the mean±SD. * *P*<0.05, ** *P*<0.01, *** *P*<0.001.

We first used GEPIA database ( http://gepia.cancer-pku.cn/) to analyze the most differentially expressed survival genes (MDSGs) in datasets of esophagus cancer patients. We screened the top 100 MDSGs from overall survival (OS) and disease recurrence-free survival (DFS) groups, of which the cutoff rate was chosen as median ( Supplementary Tables S1 and S2). The results showed that the intersection set on the first 100 MDSGs of OS and DFS, and two MDSGs, i.e., *RBBP7* and *CTD-2033A16*. *2*, showed significant differences between OS and DFS groups ( Figure 1A). A detailed analysis of RBBP7 in the OS group and DFS group on the GEPIA website indicated that RBBP7 is significantly correlated with the OS rate and DFS rate of esophagus cancer ( Figure 1B,C). High RBBP7 expression is correlated with low OS rate and DFS rate. We analyzed the gene expression of RBBP7 in esophagus cancer and normal tissues on the GEPIA website and found that RBBP7 is highly expressed in tumor tissues ( Figure 1D). Next, we further analyzed the relationship between RBBP7 expression and the cancer stages of the individual or TP53 mutation in esophagus cancer patients in the TCGA database on the UALCAN website ( http://ualcan.path.uab.edu/). The analysis showed that RBBP7 expression is significantly higher in cancer tissues than in normal tissues of esophagus cancer patients ( Figure 1E, *P*<0.01), and the patients with high tumor stage exhibits higher RBBP7 expression than those with low stage ( Figure 1F, *P*<0.01). In addition, RBBP7 expression is also higher in esophagus cancer patients with TP53 mutations than in those without TP53 mutations ( Figure 1G, *P*<0.05). On the Kaplan Meier-plotter website ( https://kmplot.com/analysis/), prognostic analysis indicated that esophagus cancer patients with high expression of RBBP7 have a poor OS rate ( Figure 1H).

### RBBP7 promotes esophagus cancer cell proliferation

**Figure 2:**
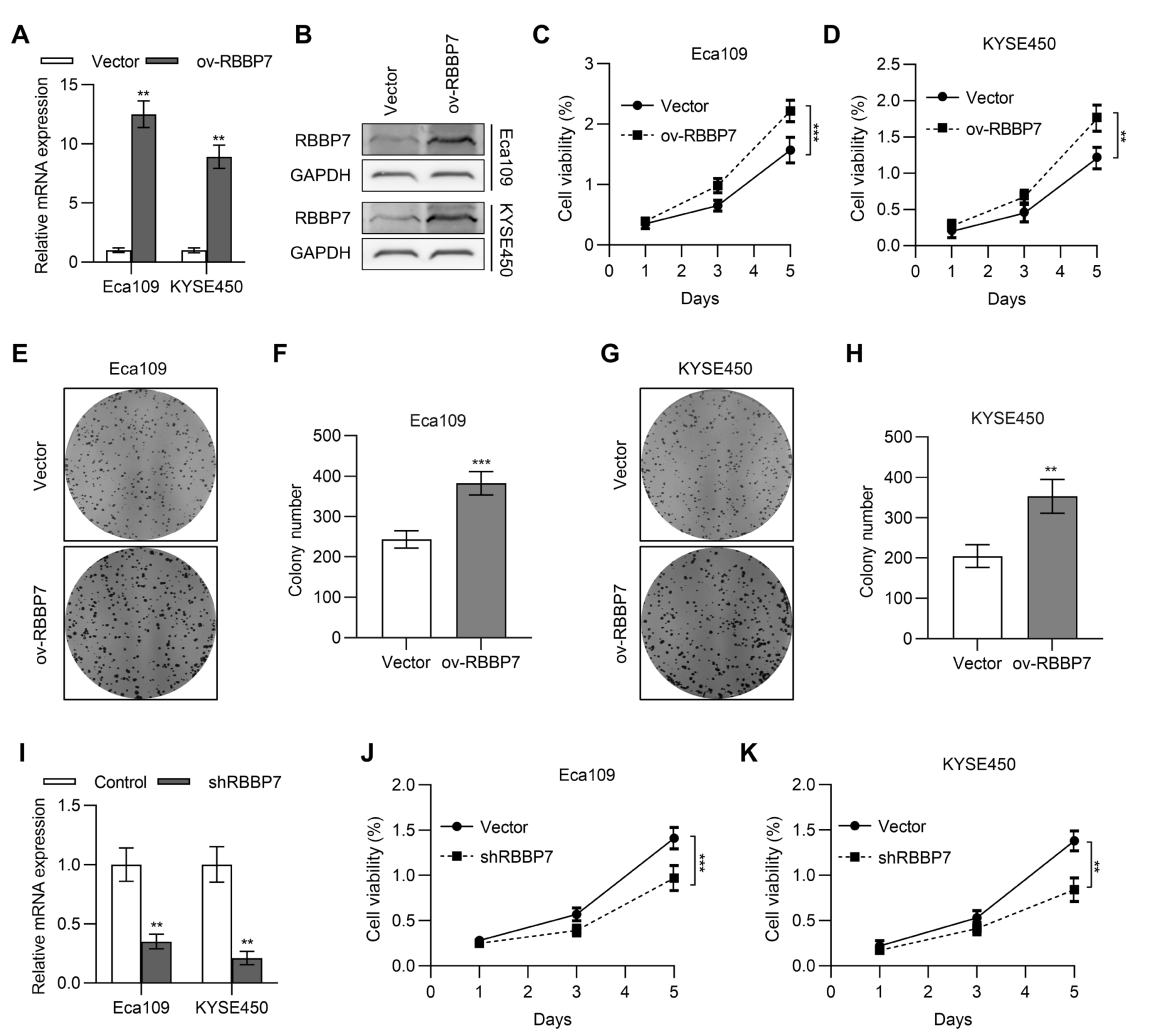
Figure2 **RBBP7 promotes esophagus cancer cells proliferation** (A,B) The mRNA (A) and protein (B) levels of RBBP7 in Eca109 and KYSE450 cells transfected with RBBP7 expression plasmid were determined by qPCR and western blot analysis. (C) Cell viability of Eca109 cells transfected with RBBP7 expression plasmid was determined by MTT assay. (D) Cell viability of KYSE450 cells transfected with RBBP7 expression plasmid was determined by MTT assay. (E) A plate colony formation assay was used to detect the colony formation rate of Eca109 cells transfected with RBBP7 expression plasmid or control plasmid. (F) The statistical result of E. (G) A plate colony formation assay was used to detect the colony formation rate of KYSE450 cells transfected with RBBP7 expression plasmid or control plasmid. (H) The statistical result of G. (I) The mRNA levels of RBBP7 in Eca109 and KYSE450 cells transfected with RBBP7 shRNA was determined by qPCR. (J–K) Cell viability of Eca109 (J) and KYSE450 (K) cells transfected with RBBP7 shRNA was determined by MTT assay. Data are shown as the mean±SD. ** *P*<0.01, *** *P*<0.001.

In order to determine the role of RBBP7 in cell function, we first overexpressed RBBP7 in Eca109 and KYSE450 esophageal cancer cell lines. qPCR and western blot analysis results verified the efficiency of RBBP7 overexpression ( Figure 2A,B). Then, the viabilities of Eca109 and KYSE450 cells transfected with RBBP7 plasmids were determined by MTT assay. We observed that the cell viability was higher in the RBBP7 overexpression group than in the vector group in both Eca109 and KYSE450 esophageal cancer cell lines ( Figure 2C,D). We also found that overexpression of RBBP7 could promote the proliferation of Eca109 and KYSE450 esophageal cancer cells ( Figure 2E–H) in colony formation experiments. At the same time, RBBP7 RNA interference were introduced in Eca109 and KYSE450 cells ( Figure 2I). RBBP7 knockdown could inhibit the viability of Eca109 and KYSE450 cells ( Figure 2J,K) as expected. These results verified that overexpression of RBBP7 could promote the proliferation of Eca109 and KYSE450 esophageal cancer cells.

### RBBP7 promotes cell stemness in esophagus cancer

**Figure 3:**
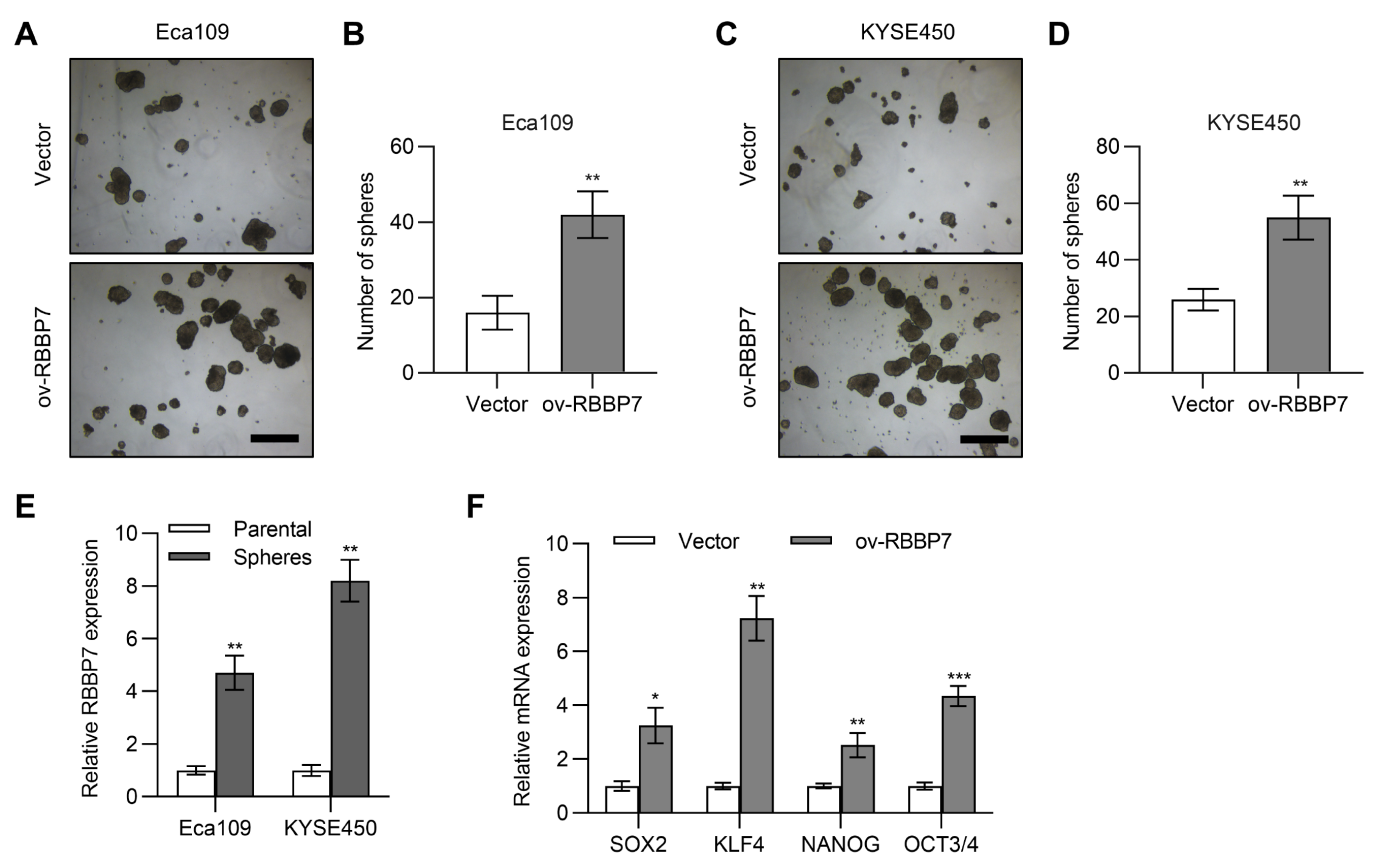
Figure3 **RBBP7 promotes cell stemness in esophagus cancer** (A) Images of tumorsphere formation assay of Eca109 cells transfected with RBBP7 expression plasmid. Scale bar: 300 μm. (B) Analyses of tumorsphere formation results of A. (C) Images of tumorsphere formation assay of KYSE450 cells transfected with RBBP7 expression plasmid. Scale bar: 300 μm. (D) Analyses of tumorsphere formation results of C. (E) qPCR results of RBBP7 levels in parental cells and spheres of Eca109 or KYSE450. (F) qPCR results of stemness markers in Eca109 cells transfected with RBBP7 expression plasmid. Data are shown as the mean±SD. * *P*<0.05, ** *P*<0.01, *** *P*<0.001.

Next, we tested whether RBBP7 plays a role in esophageal cancer stem cells. Through sphere formation assay, we found that overexpression of RBBP7 significantly increased the sphere formation ability of Eca109 and KYSE450 esophageal cancer cells ( Figure 3A–D and Supplmentary Figure S2). Next, we used qPCR to detect the expression of RBBP7 in Eca109 and KYSE450 cells and in their corresponding sphere cells. The results showed that expression of RBBP7 in the sphere cells was significantly higher than that in normal adherent cells ( Figure 3E). The stem cell markers were detected by qPCR in Eca109 cells, and the results showed that overexpression of RBBP7 upregualted the expressions of esophageal cancer stem cell markers, including SOX2, KLF4, NANOG, and OCT3/4 ( Figure 3F). These results showed that RBBP7 could promote esophageal cancer cell stemness.

### Hypoxia induces RBBP7 expression

**Figure 4:**
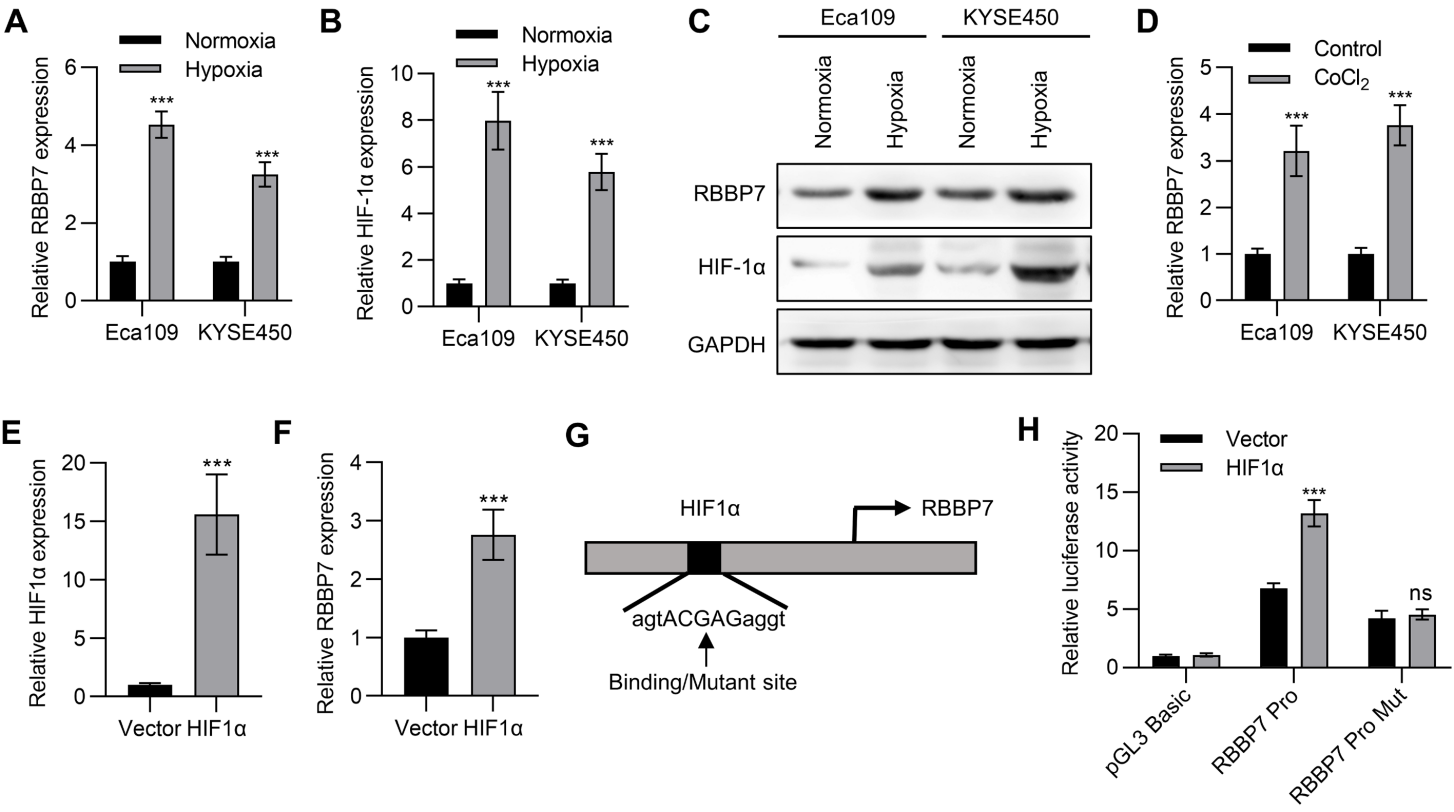
Figure4 **Hypoxia induces RBBP7 expression** (A,B) The expression of RBBP7 (A) or HIF-1α (B) in Eca109 or KYSE450 cells cultured under normoxia or hypoxia conditions was determined by qPCR. (C) The protein expressions of RBBP7 and HIF-1α in Eca109 or KYSE450 cells cultured under normoxia or hypoxia conditions were determined by western blot analysis. (D) The expression of RBBP7 in Eca109 or KYSE450 cells treated with CoCl _2_ was determined by qPCR. (E) The expression of HIF1α in Eca109 cells transfected with HIF1α expression plasmid was determined by qPCR. (F) The expression of RBBP7 in Eca109 cells transfected with HIF1α expression plasmid was determined by qPCR. (G) The schematic diagram of the HIF1α binding site on the RBBP7 promoter region. (H) Luciferase reporter assay of RBBP7 promoter-reporter in Eca109 cells transfected with HIF1α expression plasmid. Data are shown as the mean±SD. *** *P*<0.001. ns, not significant.

Next, we try to study whether hypoxia could induce RBBP7 expression. Eca109 and KYSE450 cells were cultured under normoxia and hypoxia, respectively, and the expression of RBBP7 and HIF-1α was detected by qPCR and western blot analysis. The results illustrated that the relative mRNA and protein levels of RBBP7 and HIF-1αunder hypoxia were significantly higher than those under normoxia in both Eca109 and KYSE450 cells ( Figure 4A–C). Then, Eca109 and KYSE450 cells were also treated with CoCl _2_, which could simulate a hypoxic environment. RT-PCR detection suggested that CoCl _2_ treatment significantly promoted the expression of RBBP7 ( Figure 4D). Furthermore, we overexpressed HIF1α in Eca109 cells ( Figure 4E), and found that HIF1α overexpression also promoted the mRNA expression of RBBP7 ( Figure 4F). We used rVista software ( https://rvista.dcode.org/) to predict the transcription factor binding sites in the promoter region of RBBP7, and did find a binding site for HIF1α ( Figure 4G). Luciferase report experiment verified that HIF1α overexpression could promote the fluorescent activity of the reporter vector containing the RBBP7 promoter, and after the HIF1α binding site was mutated, this promotion disappeared ( Figure 4H). These data indicated that HIF1α indeed regulates the expression of RBBP7 through transcriptional regulation.

### RBBP7 promotes CDK4 expression

**Figure 5:**
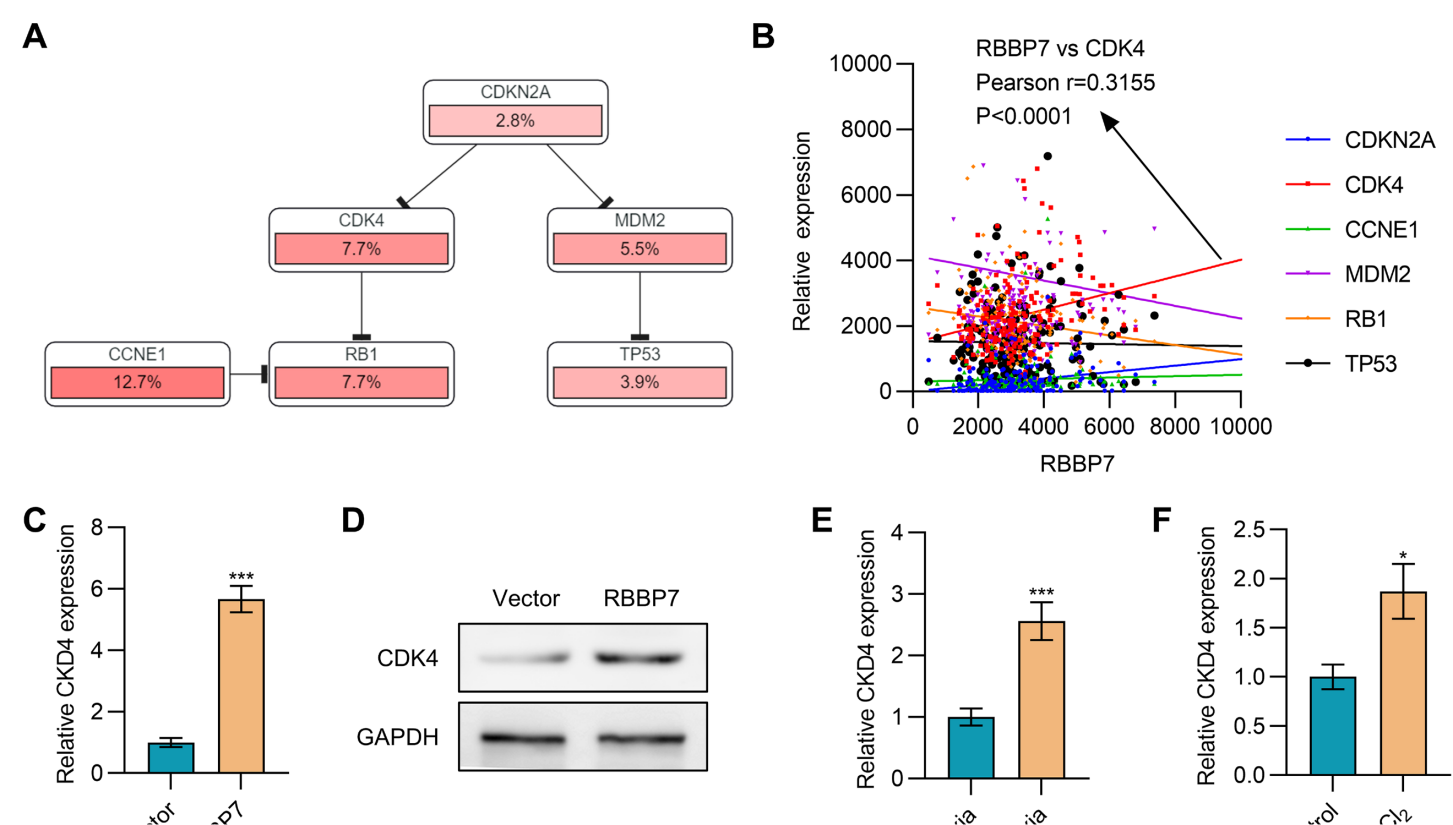
Figure5 **RBBP7 promotes CDK4 expression**(A) RBBP7 is involved in the cell cycle signal pathway. The data was analyzed in TCGA esophagus cancer (TCGA, PanCancer Atlas) using cBioportal. (B) The correlation between the expression levels of RBBP7 and cell cycle-associated genes was analyzed in the TCGA esophagus cancer (TCGA, PanCancer Atlas) dataset. (C,D) The expression of CDK4 in Eca109 cells transfected with RBBP7 expression plasmid was determined by qPCR (C) or western blot analysis (D). (E) The expression of CDK4 in Eca109 cells cultured under normoxia or hypoxia conditions was determined by qPCR. (F) The expression of CDK4 in Eca109 cells treated with CoCl _2_ was determined by qPCR. Data are shown as the mean±SD. * *P*<0.05, *** *P*<0.001.

Through the analysis of RBBP7-involved signaling pathways in the TCGA esophageal cancer patient database using cBioportal ( http://cbioportal.org), we found that RBBP7 is closely correlated with some cell cycle signaling pathways ( Figure 5A). Further results showed that RBBP7 and CDK4 exhibit a significant positive correlation in esophageal cancer patients ( *r*=0.3155, *P*<0.0001; Figure 5B), indicating that RBBP7 might regulate the expression of CDK4. Overexpression of RBBP7 in Eca109 cells indeed significantly promoted the mRNA and protein levels of CDK4 ( Figure 5C,D). We further tested whether hypoxia could also induce the expression of CDK4. The results showed that Eca109 cells cultured under hypoxia condition or CoCl _2_-induced oxygen deprivation condition could promote the expression of CDK4 ( Figure 5E,F). These data indicated that hypoxia could induce the expression of RBBP7 to further upregulate CDK4, thereby promoting the malignant process of esophageal cancer.

## Discussion

Esophageal cancer is a malignant tumor with a very high fatality rate. However, the pathogenic mechanism is not fully understood [ 1– 3]. It is vital to discover novel biomarkers and therapeutic targets. In this study, bioinformatics information derived from GEPIA and UALCAN is integrated to screen the survival-related and tumor stage-relevant genes. RBBP7 was identified and experimentally verified to be directly targeted by HIF1α to up-regulated CDK4 in hypoxia conditions to promote viability, proliferation, and stemness of esophageal cancer cells. Our data indicate that hypoxia promotes the progression of esophageal cancer through an epigenetic mechanism mediated by RBBP7. Our framework methodology is a valuable tool to disclose survival-related and tumor stage-relevant genes attributing to a particular tumor.

Hypoxia is a hallmark and a key physiological feature of esophageal cancer. However, a major challenge remains in screening tractable molecular targets that hypoxic cancer cells depend on for survival. The identified regulation of RBBP7 by HIF1α can link the hypoxia microenvironment with epigenetic regulation, which may pave the way for hypoxia-induced epigenetic research.

As an epigenetic factor, RBBP7 binds to histone deacetylase 1 (HDAC1) and specificity protein 1 (Sp1) [18] to exert complicated and contradictory functions in cancer development. RBBP7 could inhibit tumor growth by regulating c-Jun N-terminal kinase (JNK) signal transduction and function as a tumor suppressor gene [19]. In esophageal cancer, one study reported that RBBP7 overexpression or knockdown could significantly promote or inhibit the migration and invasion of esophageal squamous cell carcinoma (ESCC) cells without affecting apoptosis or tumor growth [20]. While in another study, RBBP7 was found to promote esophageal cancer cell proliferation [21], consistent with our observation. Whether such discrepancy is attributed to the different cell lines utilized or the influence of the hypoxia microenvironment warrants further analysis.

Nevertheless, there are still some limitations in this study. RBBP7 is a ubiquitously-expressed nuclear protein and belongs to a highly conserved subfamily of WD-repeat proteins, which may bind directly to Rb protein to further increase CDK4 expression [ 22, 23]. Whether some unknown mechanism is also involved needs to be further analyzed in detail. RBBP7 can up-regulate the colony formation and the relative expression of stemness markers in esophageal cancer cells, and the role of RBBP7 in the tumor stem cells may be an exciting research field in the future.

In summary, our investigation demonstrates that the hypoxia microenvironment can induce HIF1α-dependent RBBP7-mediated up-regulation of CDK4 to promote the progression of esophageal cancer. Hypoxia-induced HIF1α could up-regulate RBBP7/CDK4 to promote esophageal cancer progression, which might be considered as a treatment option.

## Supporting information

240Supplementary

## Supplementary Data

Supplementary Data is available at *Acta Biochimica et Biphysica Sinica* online.
