# Supplementary material for: Hypoxia-induced RBBP7 promotes esophagus cancer progression by inducing CDK4 expression: RBBP7 promotes esophagus cancer progression
Source: Acta Biochim Biophys Sin (Shanghai). 2022 Jan 19;54(2):179–86. doi: 10.3724/abbs.2021027 (PMC9909297; doi:10.3724/abbs.2021027)
Supplement: 240Supplementary [file 240Supplementary.pdf]

Supplementary materials

A

Eca109

Amelogenin: X
CSF1PO: 9,10
D13S317: 12,13.3
D16S539: 9,10
D5S818: 11,12
D7S820: 8,12
THO1: 7
TPOX: 8,12
vWA: 16,18

KYSE450

Amelogenin: X
CSF1PO: 11,12
D3S1358: 18
D5S818: 10,13
D7S820: 11,12
D8S1179: 10
D13S317: 11
D16S539: 9
D18S51: 13
D21S11: 30
FGA: 24
Penta D: 9,10
Penta E: 11,16
TH01: 8,9
TPOX: 8
vWA: 14

B

Eca109 **KYSE450**

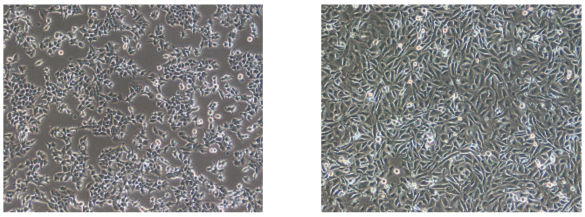

Figure S1. STR profile (A) and the image of Eca109 and KYSE450.

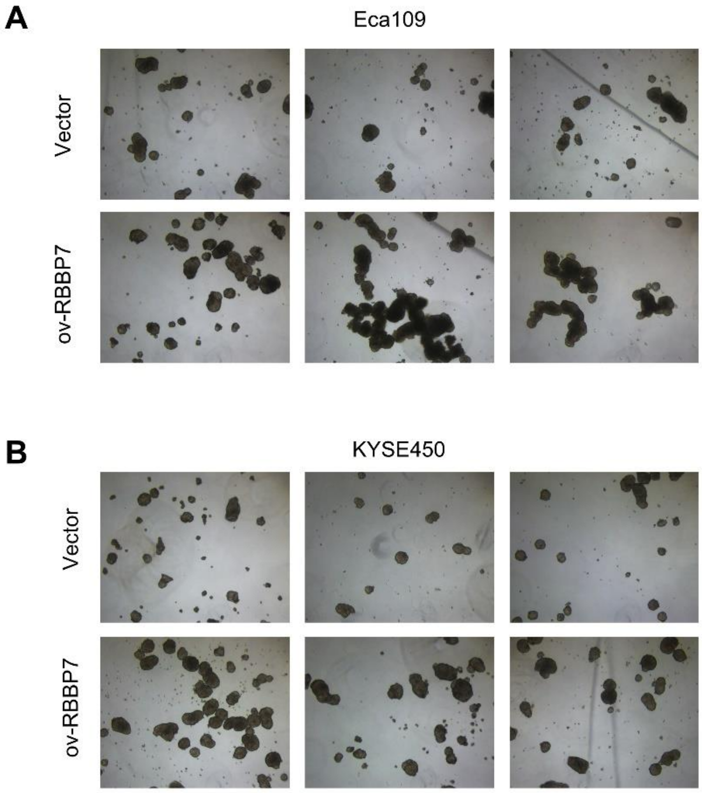

Figure S2. (A-B) The independent repeated spheres image of Fig. 3A and Fig. 3C.

Table S1 The top 100 list of most differential overall survival (OS) genes in esophageal carcinoma in GEPIA dataset.

Gene Symbol	Gene ID	P-Value (Survival OS)
RP11-103H7.5	ENSG00000254010.1	6.51E-05
INGX	ENSG00000243468.5	2.46E-04
ZNF586	ENSG00000083828.15	2.61E-04
CFAP52	ENSG00000166596.14	3.84E-04
KIAA1919	ENSG00000173214.5	5.92E-04
IGBP1-AS1	ENSG00000203588.3	6.21E-04
CDC42P5	ENSG00000253439.1	6.28E-04
DNAJC28	ENSG00000177692.11	6.70E-04
UGDH-AS1	ENSG00000249348.1	7.05E-04
AC002066.1	ENSG00000237813.3	1.02E-03
AC087501.1	ENSG00000225751.2	1.12E-03
RN7SL144P	ENSG00000242559.3	1.20E-03
KANTR	ENSG00000232593.6	1.20E-03
MT1E	ENSG00000169715.14	1.30E-03
ERVV-1	ENSG00000269526.1	1.32E-03
PDCD5P1	ENSG00000255909.1	1.39E-03
ZCWPW1	ENSG00000078487.17	1.39E-03
UBE2B	ENSG00000119048.7	1.44E-03
ATP6V0D2	ENSG00000147614.3	1.61E-03
NYAP1	ENSG00000166924.8	1.63E-03
UCHL3	ENSG00000118939.17	1.72E-03

EFNA1	ENSG00000169242.11	1.77E-03
GS1-421I3.5	ENSG00000277663.1	1.83E-03
AC004383.3	ENSG00000226107.1	1.88E-03
CTD-2319I12.2	ENSG00000267416.1	1.88E-03
HNRNPMP1	ENSG00000259335.1	1.91E-03
FAM189A2	ENSG00000135063.17	1.94E-03
TOM1L2	ENSG00000175662.17	1.95E-03
ACTN3	ENSG00000248746.5	2.16E-03
RP11-552F3.9	ENSG00000267801.1	2.21E-03
IGBP1	ENSG00000089289.15	2.34E-03
EVA1C	ENSG00000166979.12	2.38E-03
AC005776.1	ENSG00000270802.2	2.45E-03
APOOL	ENSG00000155008.13	2.45E-03
LAMP2	ENSG00000005893.15	2.47E-03
COL25A1	ENSG00000188517.14	2.53E-03
MCTS1	ENSG00000232119.7	2.54E-03
TERT	ENSG00000164362.18	2.56E-03
SLC25A1P1	ENSG00000254479.1	2.59E-03
MS4A15	ENSG00000166961.14	2.65E-03
GNL3L	ENSG00000130119.15	2.77E-03
CTA-351J1.1	ENSG00000226624.1	2.78E-03
TSPYL6	ENSG00000178021.10	2.79E-03
RBBP7	ENSG00000102054.17	2.85E-03
CTA-221G9.10	ENSG00000271181.1	2.91E-03

RP11-244H3.4	ENSG00000271741.1	2.98E-03
RP11-137H2.4	ENSG00000226659.1	3.16E-03
RP11-473O4.3	ENSG00000253720.1	3.20E-03
OVOL1	ENSG00000172818.9	3.22E-03
RP11-848P1.3	ENSG00000265743.1	3.24E-03
RNVU1-4	ENSG00000277610.1	3.33E-03
CTC-459M5.1	ENSG00000250882.1	3.54E-03
STK16	ENSG00000115661.13	3.61E-03
PKD2L1	ENSG00000107593.16	3.65E-03
SP140L	ENSG00000185404.16	3.66E-03
RP3-473L9.4	ENSG00000257595.2	3.71E-03
CCBL2	ENSG00000137944.16	3.73E-03
B3GNT2	ENSG00000170340.10	3.75E-03
ERO1LB	ENSG00000086619.13	4.06E-03
PBDC1	ENSG00000102390.10	4.27E-03
FTLP14	ENSG00000260459.2	4.27E-03
NKX6-1	ENSG00000163623.9	4.35E-03
PHF8	ENSG00000172943.18	4.38E-03
RN7SL364P	ENSG00000243560.3	4.40E-03
PMS2P1	ENSG00000078319.8	4.52E-03
RP3-368A4.6	ENSG00000271533.1	4.55E-03
RP5-851M4.1	ENSG00000277901.1	4.62E-03
AGAP3	ENSG00000133612.18	4.63E-03
RP11-480I12.9	ENSG00000243113.1	4.75E-03

RPL34-AS1	ENSG00000234492.4	4.82E-03
OXCT2	ENSG00000198754.5	4.90E-03
RP11-481A20.10	ENSG00000254507.2	4.96E-03
KCTD8	ENSG00000183783.6	5.03E-03
ASRGL1	ENSG00000162174.12	5.15E-03
DLK2	ENSG00000171462.14	5.30E-03
RP13-36G14.4	ENSG00000270223.1	5.35E-03
AXIN1	ENSG00000103126.14	5.38E-03
RP11-6B6.3	ENSG00000236942.1	5.39E-03
CLEC18A	ENSG00000157322.16	5.54E-03
NEURL1	ENSG00000107954.10	5.56E-03
RP11-311F12.2	ENSG00000264785.1	5.62E-03
LCNL1	ENSG00000214402.6	5.66E-03
U1	ENSG00000206828.1	5.69E-03
CXorf23	ENSG00000173681.16	5.72E-03
LARP1P1	ENSG00000217159.2	5.75E-03
PIGA	ENSG00000165195.13	5.83E-03
U1	ENSG00000274210.1	5.84E-03
TMEM74B	ENSG00000125895.5	5.94E-03
PPP2R4	ENSG00000119383.19	5.98E-03
IMPA1P	ENSG00000251521.2	6.00E-03
GCAT	ENSG00000100116.16	6.01E-03
TAF9B	ENSG00000187325.4	6.04E-03
CTAGE15	ENSG00000271079.1	6.10E-03

RP11-71H17.1	ENSG00000242199.1	6.11E-03
RP11-33N14.5	ENSG00000276292.1	6.14E-03
RP11-669N7.2	ENSG00000249196.6	6.17E-03
CTD-2033A16.2	ENSG00000260290.2	6.23E-03
RP11-575F12.3	ENSG00000278266.1	6.27E-03
AC093609.1	ENSG00000230587.1	6.31E-03
TJAP1	ENSG00000137221.14	6.40E-03

Table S2 The top 100 list of most differential disease free survival (RFS) genes in esophageal carcinoma in GEPIA dataset.

Gene Symbol	Gene ID	P-Value (Survival RFS)
TXNP6	ENSG00000234036.4	6.56E-05
PCF11	ENSG00000165494.10	1.65E-04
LSINCT5	ENSG00000281560.1	1.69E-04
HMGB1P8	ENSG00000259699.2	2.10E-04
GFAP	ENSG00000131095.11	2.11E-04
SUGT1	ENSG00000165416.14	3.34E-04
CALM2	ENSG00000143933.16	3.56E-04
RP11-159G9.5	ENSG00000229729.6	4.61E-04
TMED10P2	ENSG00000239405.1	5.10E-04
USP8P1	ENSG00000214892.4	5.62E-04
CDKN3	ENSG00000100526.19	5.78E-04
GSTM1	ENSG00000134184.12	6.52E-04
MTCP1	ENSG00000214827.9	6.75E-04
SNRPFP1	ENSG00000231878.1	7.09E-04
EPHA8	ENSG00000070886.10	7.63E-04
CLK2P1	ENSG00000232553.3	7.69E-04
TCL1A	ENSG00000100721.10	8.28E-04
RP11-5P18.1	ENSG00000234607.2	8.65E-04
SPP1	ENSG00000118785.13	8.67E-04
CTD-2033A16.2	ENSG00000260290.2	9.44E-04
WNT3	ENSG00000108379.9	1.03E-03

DNAJC15	ENSG00000120675.5	1.03E-03
NPM1P27	ENSG00000249353.2	1.09E-03
INO80B-WBP1	ENSG00000274049.4	1.29E-03
INTS6P1	ENSG00000250492.1	1.30E-03
RGPD8	ENSG00000169629.11	1.33E-03
FBXW10	ENSG00000171931.12	1.34E-03
PPP2R5C	ENSG00000078304.19	1.47E-03
ASPRV1	ENSG00000244617.2	1.52E-03
RPL7P23	ENSG00000244363.3	1.58E-03
FKBP3	ENSG00000100442.10	1.70E-03
RP11-567M21.3	ENSG00000255057.1	1.74E-03
RP3-388N13.5	ENSG00000264448.3	1.75E-03
ASCL5	ENSG00000232237.3	1.76E-03
TMX2	ENSG00000213593.9	1.76E-03
EIF5	ENSG00000100664.10	1.78E-03
LSM3P2	ENSG00000257847.1	1.83E-03
AC004490.1	ENSG00000267122.1	2.02E-03
SATB2	ENSG00000119042.16	2.04E-03
RPPH1	ENSG00000259001.3	2.12E-03
FAM13B	ENSG00000031003.10	2.20E-03
ZNF614	ENSG00000142556.18	2.22E-03
CAPN3	ENSG00000092529.22	2.24E-03
ITGA11	ENSG00000137809.16	2.25E-03
RP11-403A21.1	ENSG00000265752.2	2.26E-03

CIDEA	ENSG00000176194.17	2.30E-03
PTPRO	ENSG00000151490.13	2.32E-03
CARS2	ENSG00000134905.16	2.40E-03
NTM	ENSG00000182667.14	2.43E-03
RP11-252I13.1	ENSG00000248827.1	2.44E-03
RBBP7	ENSG00000102054.17	2.50E-03
SPRYD7	ENSG00000123178.14	2.50E-03
ZNF613	ENSG00000176024.16	2.66E-03
MOB1B	ENSG00000173542.8	2.66E-03
API5P1	ENSG00000234558.1	2.69E-03
SLC22A7	ENSG00000137204.14	2.70E-03
RP5-890E16.2	ENSG00000263412.1	2.71E-03
EEF1DP3	ENSG00000229715.4	2.85E-03
RP5-908M14.5	ENSG00000233017.2	2.92E-03
MBTPS2	ENSG00000012174.11	2.96E-03
DNAAF1	ENSG00000154099.17	3.04E-03
uc_338	ENSG00000274845.1	3.15E-03
TTC8	ENSG00000165533.18	3.25E-03
RPS28	ENSG00000233927.4	3.27E-03
CTD-2192J16.11	ENSG00000230310.1	3.33E-03
ESM1	ENSG00000164283.12	3.34E-03
RP11-446E9.1	ENSG00000236814.1	3.34E-03
HSD17B8	ENSG00000204228.3	3.35E-03
RING1	ENSG00000204227.4	3.51E-03

RPL5P1	ENSG00000241061.3	3.53E-03
RAB15	ENSG00000139998.14	3.54E-03
EIF1	ENSG00000173812.10	3.60E-03
BGN	ENSG00000182492.15	3.62E-03
USP12PX	ENSG00000226081.2	3.67E-03
HIST1H2BE	ENSG00000274290.1	3.67E-03
LINC00888	ENSG00000240024.5	3.68E-03
CAPZA3	ENSG00000177938.4	3.72E-03
RP5-857K21.7	ENSG00000229344.1	3.72E-03
TCEAL4	ENSG00000133142.17	3.78E-03
RP1-118J21.5	ENSG00000236546.1	3.79E-03
ANKRD18B	ENSG00000230453.9	3.83E-03
CTD-2659N19.9	ENSG00000267212.1	3.87E-03
TARID	ENSG00000227954.6	3.90E-03
RNU11	ENSG00000270103.3	3.92E-03
SALL4	ENSG00000101115.12	4.07E-03
ST13P4	ENSG00000232150.3	4.09E-03
PCDHGA9	ENSG00000261934.2	4.15E-03
HNRNPA3P10	ENSG00000257851.2	4.24E-03
RP11-20B7.1	ENSG00000242741.1	4.25E-03
RP11-314B1.2	ENSG00000273301.1	4.28E-03
EIF2S2P4	ENSG00000128692.8	4.29E-03
ZNF433	ENSG00000197647.11	4.29E-03
RP11-307L3.2	ENSG00000233846.2	4.38E-03

QDPR	ENSG00000151552.11	4.39E-03
AC005251.3	ENSG00000219451.3	4.55E-03
CERKL	ENSG00000188452.13	4.58E-03
TTC34	ENSG00000215912.10	4.60E-03
TMEM258	ENSG00000134825.13	4.61E-03
SAP18	ENSG00000150459.12	4.69E-03
RANP1	ENSG00000236603.2	4.80E-03
